# CRISPR-Cas System Is an Effective Tool for Identifying Drug Combinations That Provide Synergistic Therapeutic Potential in Cancers

**DOI:** 10.3390/cells12222593

**Published:** 2023-11-09

**Authors:** Yuna Kim, Hyeong-Min Lee

**Affiliations:** Department of Computational Biology, St. Jude Research Hospital, Memphis, TN 38105, USA; ykim1@stjude.org

**Keywords:** CRISPR-Cas, drug combination, synergy, neuroblastoma, pediatric cancer

## Abstract

Despite numerous efforts, the therapeutic advancement for neuroblastoma and other cancer treatments is still ongoing due to multiple challenges, such as the increasing prevalence of cancers and therapy resistance development in tumors. To overcome such obstacles, drug combinations are one of the promising applications. However, identifying and implementing effective drug combinations are critical for achieving favorable treatment outcomes. Given the enormous possibilities of combinations, a rational approach is required to predict the impact of drug combinations. Thus, CRISPR-Cas-based and other approaches, such as high-throughput pharmacological and genetic screening approaches, have been used to identify possible drug combinations. In particular, the CRISPR-Cas system (Clustered Regularly Interspaced Short Palindromic Repeats) is a powerful tool that enables us to efficiently identify possible drug combinations that can improve treatment outcomes by reducing the total search space. In this review, we discuss the rational approaches to identifying, examining, and predicting drug combinations and their impact.

## 1. Introduction

Neuroblastoma (NB) is one of the pediatric cancers arising from immature nerve cells, most commonly from the adrenal glands [1]. NB accounts for about 10–15% of deaths in the childhood population [2]. According to the American Cancer Society, the 5-year survival rate for high-risk NB (metastatic) patients is around 50%. Although the death rate in NB patients is widely different due to various factors, such as the stage of the cancer at diagnosis, the age of the patient, and the treatment options available, the 5-year survival rate of 50% seems stagnant despite numerous efforts to improve it [3]. Thus, there are unmet needs in the development of effective therapeutic interventions targeting NB. Indeed, many research groups have taken advantage of multiple approaches to develop effective therapeutics as several approaches hold promise.

## 2. Approaches to Develop Therapeutics for Neuroblastoma and Other Cancers

Those approaches include drug repurposing, targeted therapy, immunotherapy, and precision (or personalized) medicine, but are not limited to them. Drug repurposing is the process of changing the intended use of an already existing drug. In other words, it aims to identify new indications for existing drugs [4,5,6]. Drug repurposing can accelerate therapeutic development and gain improved modality in cancer treatment [6] because of several advantages. First, safety and toxicity are already established in existing drugs, which enable us to accelerate the development process of new cancer treatments. Second, drug repurposing can provide new treatment options for cancer patients who have certain drug resistance or show a lack of effective treatments. Third, repurposing can lead to the discovery of new mechanisms of action for existing drugs, which can be used to develop new therapies. In addition, drug repurposing can be used in a combination manner to identify novel and more effective cancer therapeutics [7,8]. New therapeutics that are more effective than existing treatments can be identified by screening existing drugs for anti-cancer activities, followed by testing them in combination with other anti-cancer agents. For example, a combination of cisplatin and irinotecan was used for non-small cell lung cancer (NSCLC) [9], but it was redirected to treat gastrointestinal malignancy [10,11]. The targeted therapy approach uses drugs aiming at specific molecular targets or pathways that are involved in the growth and metastasis of cancer cells [12,13,14]. While traditional chemotherapy can non-specifically damage the body to various degrees, targeted therapy is designed to selectively target cancer cells to minimize non-specific damages. Thus, one advantage of targeted therapy is that it often causes fewer side effects than traditional chemotherapy, although it can still cause side effects. Several types of targeted therapies have been proposed. For example, drugs targeting specific receptor tyrosine kinases, such as ALK [15,16,17,18,19] and RET [19,20,21,22], are currently being explored. As targeted therapy is often used to treat advanced or metastatic cancers, targeted therapy can be used alone or in combination with other treatments, such as chemotherapy and radiation. The immunotherapy approach harnesses the immune system to attack tumors [23,24]. As tumors can evade the immune system by suppressing or hiding ligands recognized by immune cells, immunotherapy using checkpoint inhibitors [25,26,27], chimeric antigen receptor T (CAR-T) cells [28,29,30], and cancer vaccines [31,32,33] facilitates the activation of the immune system to recognize and attack cancer cells. Adoptive cell transfer is also a type of immunotherapies using modified immune cells extracted from the patients themselves [34,35]. Immunotherapy has shown promising results in treating different types of cancers, especially several combination therapies that have already been approved by the FDA across different cancer types [36]. Last but not least, due to technical advancements in next-generation sequencing, precision (or personalized) medicine leverages specific genetic mutations or molecular markers that drive cancers. As a result, it allows us to tailor treatments accordingly. Molecular profiling of tumors in patients can help identify genetic factors affecting cancer growth and/or metastasis [37,38,39], and other factors influencing a patient’s response to treatment; this approach enables us to develop precise targeted therapy or immunotherapy, including in combination with other treatments [40].

## 3. Drug Combinations

**Figure 1:**
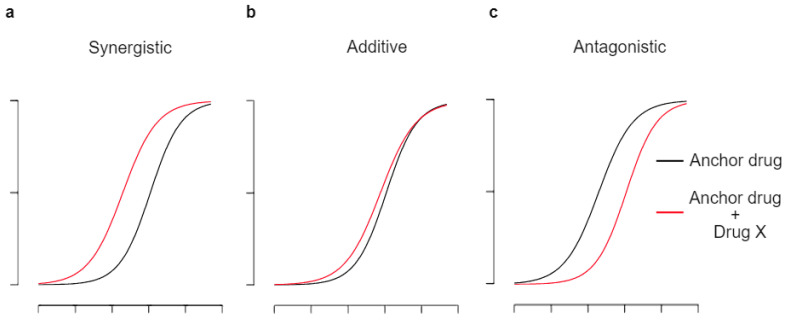
Simplified impacts of drug combination. The changes in potency between the anchor drug (black) and its combination with drug X (red) indicate synergistic (**a**), additive (**b**), and antagonistic effects (**c**) of drug combinations (x-axis: drug dose, y-axis: drug effectiveness). As the goal of using drug combinations is to improve treatment outcomes, synergistic drug combinations (**a**) are most favorable for clinical applications, while additive (**b**) or antagonistic (**c**) effects of drug combinations may not serve clinical purposes.

The approaches described above can be used alone or in combination with other treatments to enhance the effectiveness of treatments. Thus, the combination approach is thought to be a common practice in the treatment of cancers to gain better treatment outcomes compared to single approaches [41]. The rationale behind this approach is that utilizing multiple drugs with different mechanisms of action can overcome the poor outcomes of single-drug treatments (Figure 1) [42,43]. The goals of using drug combinations are to increase therapeutic effectiveness, reduce toxicity, delay the development of drug resistance, and improve outcomes in neuroblastoma (NB) and other cancer patients [44,45].

Neuroblastoma (NB) is complex and heterogeneous. Consequently, its effective treatment often necessitates a combination of drugs that possess varied mechanisms of action. A promising strategy involves pairing a targeted therapy, known as an ALK inhibitor, with chemotherapy. This combination has shown potential to enhance outcomes for patients diagnosed with ALK-positive neuroblastoma [17,18,46,47]. Specifically, a recent phase I clinical study ([47]) provided insights into the safety and potential efficacy of lorlatinib, a third-generation ALK inhibitor, both with and without chemotherapy, for the treatment of ALK-driven refractory or relapsed neuroblastoma. However, this study did not present a comprehensive comparison of monotherapy versus combination treatment. The Food and Drug Administration (FDA) has approved several drug combinations for various indications. In this context, we briefly highlight three cases pertaining to neuroblastoma and five cases related to other cancer types (Table 1). 

However, the main challenge in drug combinations is how to identify the right combinations. For example, 2-drug combinations using 600 drugs (rounded up from 568 drugs approved by the FDA in 2008–2022 [69]) yield a total of 179,700 possible combinations. If 3-drug combinations were considered, it would yield more than 30 million unique combinations. With such a vast number of possible drug combinations, it is practically impossible to evaluate all the possibilities. Even though the approved combinations (Table 1) provide therapeutic benefits, the design and selection of combinations are not straightforward, suggesting some limitations. The limitations of drug combinations include inflexible dose ratios and incompatible pharmacokinetics (PK). An inflexible dose ratio may hinder the ability to optimize the dose of each drug for individual patients. It also challenges us to design clinical trials as they assess dose-related efficacy and toxicity [70,71]. Incompatible PK gives rise to additional complications in drug combinations. Differences in the PK of each drug (e.g., different ADME (absorption, distribution, metabolism, and excretion), and different half-lives) might cause one drug to affect the other, resulting in an alteration of the desired effectiveness of the combinations [72,73]. Additionally, previous clinical protocols for the combination approach were primarily based on empirical evidence or clinical data. Even clinical data are difficult to standardize to determine the effects of drug combinations due to the diversity of patients, including their genetic alterations, the patients’ age, the stage of the cancer, and other individual health factors. Therefore, the vast number of possible drug combinations requires a rational approach for screening and predicting the effects of drug combinations.

### Approaches to Identify Drug Combinations

Identifying the right drug combination for neuroblastoma and other cancers is a complex and challenging task. To effectively identify drug combinations beyond a traditional setting (e.g., using empirical evidence or clinical data), a variety of in vitro and in silico assays have been developed to examine and predict the potential therapeutic effects of drug combinations in recent years. 

**Figure 2:**
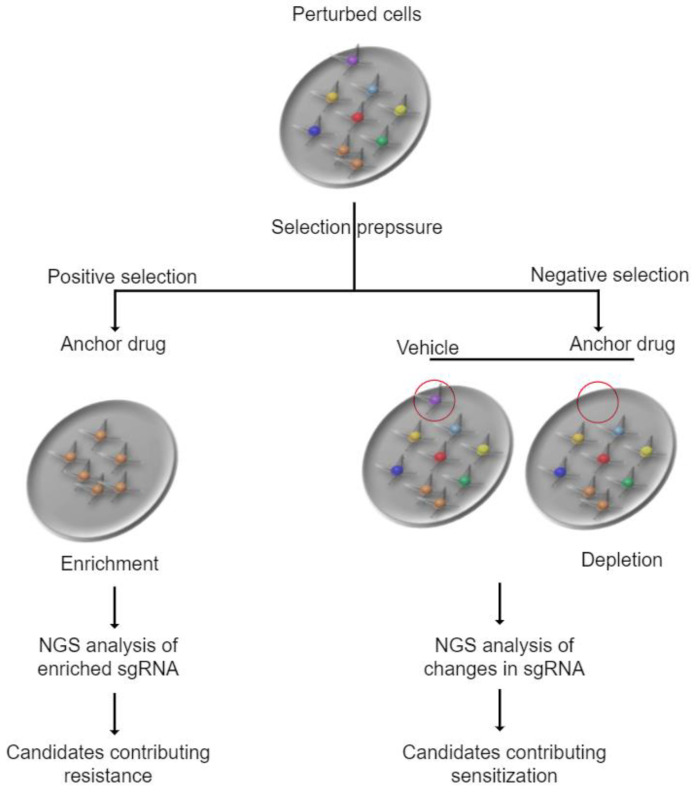
CRISPR-Cas positive and negative selection screens. In positive selection, most perturbed cells die under selection pressure, while surviving cells grow, suggesting cell enrichment. This allows us to identify candidate genes contributing to drug resistance. Negative selection enables us to perform systematic prioritization of context-specific drug combinations. In negative selection, most perturbed cells survive under selection pressure. However, selective depletion allows us to identify candidate genes contributing to sensitization (red circle). These candidate genes can be targets for drug combinations with the anchor drug.

**Figure 3:**
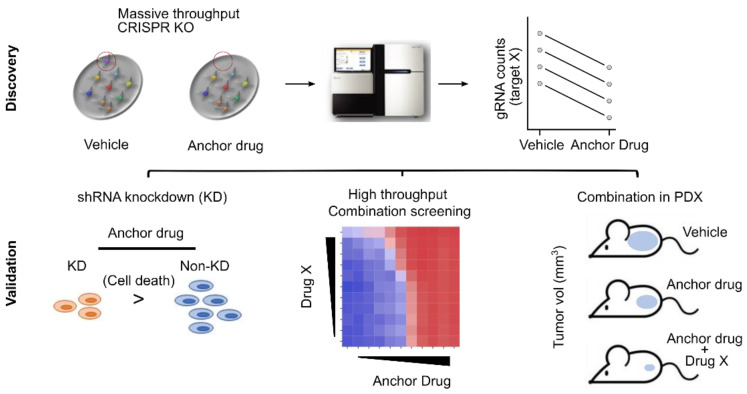
Downstream applications in CRISPR-Cas negative selection screens. Once target X, a candidate gene for drug combination with the anchor drug, is identified at the discovery stage, three downstream approaches are used to validate target X. Ideally, treatment of the target X knockdown (KD) with the anchor drug induces more cell death compared to anchor drug-treated non-KD. Second, a high-throughput combination screen of the anchor drug and drug X provides a landscape of the impact of drug combinations. Third, in vivo validation of drug combinations using PDX models provides more comprehensive information, including toxicity.

CRISPR-Cas-based approaches are being used to identify potential drug combinations for neuroblastoma and other cancers [74,75,76,77]. The CRISPR-Cas system (Clustered Regularly Interspaced Short Palindromic Repeats) is a genome editing tool that can be used to selectively disrupt or modify specific genes in cancer cells [78]. By selectively disrupting or modifying specific genes, it allows us to identify which genes are essential for the survival or growth of cancer cells and which genes can be targeted with specific drugs. Briefly, the CRISPR-Cas system is used to validate molecular target(s) by selectively disrupting the potential target gene in cancer cells and assessing the effect on cancer cell survival or growth. Once the essentiality of the target(s) in cancer cells is confirmed, it provides further evidence to support the development of specific, but different drugs aiming at the same target(s). The impact of these drug combinations could be synergistic through different mechanisms of action, such as their complementary or anti-counteractive actions (Note: The impact is not always synergistic. There are other types of combination impacts) [79]. Another direction for using the CRISPR-Cas-based approach is the use of CRISPR-Cas positive and negative selection screens (Figure 2). The selection pressure (i.e., anchor drug vs. vehicle-treated) allows us to identify resistant or sensitized genes in cancer cells. A common direction of positive selection is to discover genes that provide resistance to a given anchor drug [80,81] because it relies on cell enrichment. Due to this enrichment, the results of positive selection screens are more easily interpreted than those of negative selection screens. In negative selection screens, cell viability is one of the direct indicators of negative selection phenotypes because perturbations causing cell death are depleted over time [82,83]. When they are applied to cancer cells, they provide insights into the landscape of gene dependency in cancer cells [84,85,86]. Such methods can be used to investigate the mechanisms of drug action or to identify genes that improve the response of the anchor drug when the target genes are depleted. In particular, after the validation stages are complete (Figure 3), potentially identified genes become candidates that we expect synergism of drug combinations when the anchor drug and the drug targeting the identified gene products are combined. 

One important consideration in the selection is that when an anchor drug targets a specific molecular target (e.g., an ALK inhibitor such as ceritinib or lorlatinib), the potentially identified combination targets with an ALK inhibitor are often straightforward (ALK inhibitor + target X inhibitor), yet combination effects need to be addressed. On the other hand, some drugs show little association with their targets (i.e., some well-characterized drugs function through non-inhibitory modes, for example, etoposide is a poison forming TOP2cc [87,88], and platinum-based chemotherapies alkylate DNA to prevent DNA-associated metabolism [89,90,91]). When these drugs are used as the anchor drug, addressing their combination effects is not as straightforward as with anchor drugs that have specific targets. Nonetheless, potential combination targets can still be identified. The complexity arises, in part, because their broad mechanisms can interact with multiple pathways in the cells, making it challenging to anticipate possible outcomes. This is supported by a study that identified etoposide response modulators in acute myeloid leukemia using a CRISPR-Cas screen [92]. This suggests that it is essential to integrate CRISPR-Cas-guided screens to gain more profound insights into markers associated with drug resistance and emerging drug targets. 

Synthetic lethality screen using the CRISPR-Cas system is also a viable option to identify drug combinations [93,94,95]. Selectively disrupting two or more genes allows us to identify gene pairs that are essential for the survival of cancer cells. Once the gene pairs are identified, drug combinations targeting the proteins encoded by those genes become available to evaluate whether they have synergistic effects on cancer cells.

Additionally, other approaches to identify drug combinations are available. For instance, a high-throughput screen of combinatorial drug libraries enables us to examine potential therapeutic effects of multiple compounds. Combinatorial drug libraries are collections of large numbers of compounds synthesized and stored in mixtures [96,97]. Screening these libraries allows us to identify drug candidates that potentially have synergistic effects on cancer cells in various combinations as they take into account all possible combinations of the chemical building blocks [98]. To facilitate this process, click chemistry provides easy access to the synthesis of building blocks for novel chemical entities. Click chemistry can help structure-based compound design and improve combinatorial chemistry by selecting appropriate building blocks. Consequently, it can provide compound varieties such as chemical derivatives, analogs, pharmacophores, and drugs [99,100]. In summary, although they have their strengths and limitations (discussed below), these in vitro assays have become critical for predicting the potential therapeutic effects of combination therapy.

Since the most appropriate method depends on the context of the cancers to be addressed, here we briefly summarize several studies targeting neuroblastoma (NB). CRISPR-Cas-based approaches identify therapeutic targets in NB. CDK8, ATM, and EZH2 have been identified as potential drug targets in combinations with an MEK inhibitor, a TOP2 inhibitor, and an HDAC inhibitor, respectively, via a genome-wide CRISPR-Cas knockout screen (Table 2) [101,102,103]. A CRISPRa (CRISPR activation)-dCas screen has also identified PIM as a potential drug target in combination with an ALK inhibitor [104]. Recently, a CRISPR-Cas-based perturbational map also provides potentially selective targets in NB [105]. Last but not least, targeted therapy has identified synergism in the inhibition of CHK1 and PARP [106] (Table 2). Interestingly, the primary indications of the drugs examined in these studies were developed for multiple solid tumors and other diseases. For example, trametinib was developed for melanoma [107] and was redirected to treat NB in combination with BI-1347, which originally targeted melanoma and breast cancers [108]. While brequinar is an immunosuppressant [109], a study provided a potential new indication for NB in combination [110] with temozolomide, which originally targeted brain cancer and skin cancer [111]. These new indications for existing drugs represent drug repurposing, which can possibly accelerate the development of effective drug combinations through validating these findings and determining the safety and efficacy in future pre/clinical studies. While these studies hold promise as they suggest the synergistic therapeutic potential of drug combination therapy for neuroblastoma, it is important to choose highly selective compounds or drugs aiming at the identified target to improve the outcomes of drug combinations. Identifying potential biomarkers of the response to a drug combination can also improve the outcomes of drug combinations [112,113]. If a specific gene expression signature predicts response to a drug combination, such information can be used for guiding treatment strategies.

## 4. Advantages and Challenges in CRISPR-Based Drug Combination

The growing evidence described above supports that a CRISPR-Cas-based approach is an effective tool for identifying potential drug combinations for neuroblastoma and other cancers. However, it clearly has both advantages and limitations. The CRISPR-Cas system allows us to perform highly precise and specific gene editing. Eventually, it can help identify specific genes or gene combinations that potentially provide therapeutic benefits when effectively disrupted. In addition, it has broad applicability, which allows us to make it a versatile tool for identifying drug targets and drug combinations. Because of the advantages of the CRISPR-based approach, it provides the opportunity to reduce the total search space (e.g., 179,700 possible combinations in two-drug combinations using 600 drugs) for rapid and systematic prioritization of context-specific drug combinations. Identifying these interactions efficiently leads to drug synergies in pediatric-specific cellular contexts that can dramatically impact patient outcomes.

On the other hand, one of the major limitations of CRISPR-Cas-based approaches is that false-negative and/or false-positive outcomes can be problematic because CRISPR-Cas knockout does not 100% reflect the pharmacological inhibition of a drug target in every case. In other words, the functional inhibition of a target via small molecules is not necessarily able to recapitulate the consequences of target deletion. One strategy to address such discrepancy is to assess small-molecule inhibitors and PROTAC (or molecular glue) aiming at the same molecular target. Although the CRISPR-Cas system is highly precise, off-target effects can be problematic because of sgRNA selectivity [114]. This can lead to unintended consequences and potentially confound the results of CRISPR-Cas screens. Even though the selectivity of sgRNAs is secured, potential synergistic dependency of genes specifically localized on the amplification regions targeted by CRISPR is not always the direct consequence of deleting the target gene because gene amplifications can lead to overexpression [115]. Since some genes show universal lethality in CRISPR-Cas knockouts but represent viable targets of small molecules (e.g., *TOP2A*), CRISPR interference screens (CRISPRi) can be an alternative strategy. CRISPRi could overcome the problem with universal lethality of knockouts because gene expression is repressed by interference, rather than by being completely knocked out. However, the results of CRISPRi should be interpreted with caution because it is not clear whether CRISPRi would phenocopy the pharmacological inhibition of the target genes. Alternatively, using more specific Cas enzymes can enhance the accuracy and efficiency of CRISPR-based approaches. Due to the risk of unintended genetic changes that raise concerns about safety and accuracy, researchers have developed an improved SpCas9 version known as eSpCas9 [116]. These enhanced variants reduce off-target effects while maintaining their effectiveness at the target sites. However, new challenges have arisen. Some of these variants have limitations in their target range and may not function optimally in certain formats, especially when assembled as ribonucleoproteins. There is also a compatibility issue with specific RNA guides, particularly 21G-sgRNAs. In response, a refined SpCas9 version, called Blackjack SpCas9, was introduced [117]. This new variant not only retains the high specificity of earlier versions but also works seamlessly with 21G-sgRNAs. Advanced iterations, referred to as Blackjack nucleases (including eSpCas9-plus and SpCas9-HF1-plus), are versatile and perform consistently, regardless of whether they are introduced as DNA or ribonucleoproteins. In summary, while the foundational Cas9 brought revolutionary changes to genome editing, its precision was questionable. However, with developments like eSpCas9 and the Blackjack series, technology is rapidly evolving towards increased safety and accuracy. As CRISPR-Cas-based approaches are typically performed in in vitro settings, this suggests that results may not be 100% transferrable to in vivo settings due to the complexity and heterogeneity of cancers. To uncover novel insights into cancer biology that may not be evident in traditional 2D systems, using more complex CRISPR screening models such as 3D culture (organoids or spheroids), PDX models, or direct application to in vivo settings may provide better insights into examining and predicting the potential therapeutic effects of drug combinations. For example, a CRISPR-Cas knockout screen in a spheroid model revealing genes that are essential for 3D growth [118] and formation [119]. Previous studies have suggested that this approach may uncover potential therapeutic targets that are more relevant for treating patients. It is undeniably important for bridging the gap between in vitro and in vivo settings; however, to our knowledge, there is no clear evidence showing that CRISPR-Cas screens in 3D models can better predict drug effects. Technically, when generating 3D models (organoids or spheroids), ensuring reproducibility and consistency in 3D culture size and density is important to obtain reliable results as this approach may limit scalability, but this can be overcome by using in vivo CRISPR-Cas screens [120,121]. Nevertheless, using complex CRISPR screening models, such as 3D culture (organoids or spheroids), PDX models, or direct application to in vivo settings (tumor mouse models or humanized mouse models), may provide better insights into examining and predicting the potential therapeutic effects of drug combinations.

Additionally, as the effect of pairs of gene knockouts are often cell line specific [122,123] in CRISPR-Cas-based approaches, expanding the study to include a larger number of cancer cell lines can facilitate the identification of drug targets that are relevant across a broader range of cancers. We recently reported a unique combination of cell line perturbations by examining all druggable gene knockouts in 18 cell lines (10 neuroblastoma cell lines, 4 non-neuroblastoma cancer cell lines, and 4 non-cancer-derived cell lines) with eight standard-of-care drugs. To our knowledge, this was the largest drug combination study ever reported. This study revealed pervasive cell line-specific chemo-sensitizing knockouts, which may explain the lack of translations of findings from previous smaller-scale combinatorial screening. However, the cancer cell lines to be examined need to be much more diverse to comprehensively identify drug targets that are relevant across a broader range of cancers. In summary, CRISPR-Cas-based approaches have both advantages and limitations when it comes to identifying drug combinations for neuroblastoma and other cancers. While the CRISPR-Cas system provides a powerful tool for precision gene editing, its use is still limited by the number of genes that can be screened and the potential for off-target effects.

## 5. Conclusions

Since neuroblastoma is a childhood cancer that can be difficult to treat, identifying effective drug combinations could improve outcomes for patients. The CRISPR-Cas system and other approaches are efficient tools for identifying potential drug targets and drug combinations. Particularly, CRISPR-Cas-based approaches can be used to screen large numbers of cancer cell lines for potential drug targets and to test the effectiveness of drug combinations as it allows a reduction of the total search space for rapid and systematic prioritization of context-specific drug combinations. While the approaches in previous studies discussed here represent important steps toward identifying effective drug combinations for neuroblastoma and other types of cancer, there is always room for improvement. Potential areas for improvement include enhancing selectivity/specificity, identifying reciprocal relationships in vitro and in vivo, expanding research to include more cell lines, validating the results in multiple orthogonal approaches, and identifying potential biomarkers of response. Further research is necessary to validate these approaches and bring them into pre/clinic trials. Nevertheless, the use of CRISPR-Cas-based approaches for identifying drug combinations shows promise in improving outcomes in cancer patients, including those with neuroblastoma.

## Figures and Tables

**Table 1:** Short examples of FDA-approved drug combinations.

Combination of Drugs	Drug 1	Primary Indication For	Drug 2	Primary Indication For	Drug 3	Primary Indication For	Combination Indication	Refs
Two drugs	Busulfan	Chronic myeloid leukemia	Melphalan HCl	Multiple cancers, including neuroblastoma			Neuroblastoma	[48,49]
	Naxitamabgqgk (Danyelza)	Neuroblastoma, osteosarcoma	GM-CSF				Neuroblastoma	[50]
Three drugs	Caborplatin	Multiple cancers	Melphalan HCl	Multiple cancers, including neuroblastoma	Etoposide phosphate	Multiple cancers, including lung	Neuroblastoma	[51,52,53]
Two drugs	Pembrolizumab (Keytruda)	Multiple cancers, including melanoma	Pt-based chemotherapy	Multiple cancers			Non-small cell lung cancer	[54,55,56,57]
	Pembrolizumab (Keytruda)		Lenvatinib (Lenvima)	Thyroid cancer			Advanced renal cell	[58,59,60]
	Encorafenib (Braftovi)	Non-small cell lung cancer, melanoma	Binimetinib (Mektovi)	Multiple cancers			Carcinoma Melanoma with BRAF mutations	[61,62]
	Encorafenib (Braftovi)		Cetuximab (Erbitux)	Bowel cancer			Metastatic colorectal cancer (CRC)	[63,64,65]
Three drugs	Tremelimum ab (Imjudo)	Hepatocellular carcinoma	Durvalummab (Imfinzi)	Non-small cell lung cancer	Pt-based chemotherapy	Multiple cancers	Non-small cell lung cancer	[66,67,68]

PT-based: Platinum-based chemotherapy. GM-CSF: Granulocyte-macrophage colony-stimulating factor. Blanks: commercial (generic) names for each drug.

**Table 2:** Synergistic therapeutic potential of drug combinations in neuroblastoma *.

Approach	Anchor Drug	Primary Target	Identified Target (IdT)	Drug for IdT	Impact of Combination	Refs
CRISPR-Cas (KO)/GW	Trametinib	MEK	CDK8	BI-1347	Synergistic **	[101]
CRISPR-Cas (KO)/GW	CX-5461	TOP2	ATM	AZD1390	Synergistic	[102]
CRISPR-Cas (KO)/GW	Panobinostat	HDAC	EZH2	GSK126	Synergistic	[103]
CRISPR-dCas-VP64/GW	Ceritinib	ALK	PIM	AZD1208	Synergistic	[104]
CRISPR-Cas (KO)/customized	Doxorubicin	TOP2	PRKDC	AZD7648	Synergistic	[105]
CRISPR-Cas (KO)/customized	JAQD1	EP300	HDAC2	Panobinostat	Synergistic	[105]
CRISPR-Cas (KO)/customized	Topotecan	TOP1	KEAP1	DMF	Synergistic	[105]
Targeted therapy	MK-8776	CHK1	PARP	Olaparib	Synergistic	[106]

KO: knockout, GW: genome-wide, dCas-VP64: CRISPR-activation screen, customized: targeting 655 non-lethal genes in a neuroblastoma cell line, CHK1: checkpoint kinase 1, MEK: mitogen-activated protein kinase, ALK: anaplastic lymphoma kinase, TOP1: topoisomerase 1, TOP2: topoisomerase 2, HDAC: histone deacetylase, EP300: E1A-associated p300 transcriptional co-activator protein, PARP: poly ADP ribose polymerase, CDK8: cyclin-dependent kinase 8, PIM: proviral insertion in murine kinase, ATM: ataxia-telangiectasia mutated kinase, EZH2: enhancer of zeste 2 polycomb repressive complex 2 subunit, PRKDC: DNA-dependent protein kinase catalytic subunit, HDAC2: histone deacetylase 2, KEAP1: kelch-like ECH-associated protein 1, MET: MNNG HOS transforming kinase, DMF: dimethyl fumarate * We are not able to introduce all valuable studies, including other types of cancers, due to limited space. ** Possibly additive effects.

## Data Availability

Not applicable.
